# Preliminary study on brain resting-state networks and cognitive impairments of patients with obstructive sleep apnea–hypopnea syndrome

**DOI:** 10.1186/s12883-022-02991-w

**Published:** 2022-12-07

**Authors:** Yaqing He, Junkang Shen, Xiang Wang, Qiaozhen Wu, Jiacheng Liu, Yiding Ji

**Affiliations:** 1 Department of Radiology, Suzhou Ninth People’s Hospital, Suzhou, China; 2 Department of Radiology, The Second Affiliated Hospital of Soochow University, Suzhou, China; 3 Department of Respiratory, Suzhou Ninth People’s Hospital, Suzhou, China; 4 Department of Radiology, The Affiliated Zhongda Hospital of Southeast University Medical School, Nanjing, China

**Keywords:** Obstructive sleep apnea, Cognitive impairment, Resting-state networks, Independent component analysis

## Abstract

**Background:**

To investigate functional changes in brain resting-state networks (RSNs) in patients with obstructive sleep apnea–hypopnea syndrome (OSAHS) and their correlations with sleep breathing disorders and neurocognitive performance.

**Methods:**

In this study, 18 OSAHS patients and 18 matched healthy controls underwent neurocognitive assessment and magnetic resonance imaging (MRI). Group-level independent component analysis (ICA) and statistical analyses were used to explore between-group differences in RSNs and the relationship between functional changes in RSNs, sleep breathing disorders and neurocognitive performance.

**Results:**

The OSAHS patients performed worse on neuropsychological tests than the healthy controls. Eight RSNs were identified, and between-group analyses showed that OSAHS patients displayed significantly decreased functional connectivity in the bilateral posterior cingulate gyri (PCC) within the default mode network (DMN), the right middle frontal gyrus (MFG) within the dorsal attention network (DAN), and the left superior temporal gyrus (STG) within the ventral attention network (VAN), and increased functional connectivity in the right superior frontal gyrus (SFG) within the salience network (SN). Further correlation analyses revealed that the average ICA z-scores in the bilateral PCC were correlated with sleep breathing disorders.

**Conclusions:**

Our findings demonstrate that the DMN, SN, DAN, and VAN are impaired during the resting state and are associated with decreased functionally distinct aspects of cognition in patients with OSAHS. Moreover, the intermittent hypoxia and sleep fragmentation caused by OSAHS are likely to be the main influencing factors.

**Supplementary Information:**

The online version contains supplementary material available at 10.1186/s12883-022-02991-w.

## Background

With the prevalence of obesity and the aging of the population, the number of patients with obstructive sleep apnea–hypopnea syndrome (OSAHS) is increasing year by year [1]. Based on the available epidemiological data, 5.7%-6.0% of adult males and 2.4%-4.0% of adult females are troubled by OSAHS [2, 3], which is often associated with cognitive impairment, such as poor concentration and memory decline, which progresses over time if not treated. Recurrent partial or complete collapse of the upper airway during sleep can lead to intermittent hypoxia, hypercapnia and sleep fragmentation, which have strong associations with cognitive decline in patients with OSAHS, especially those with neurodegenerative diseases [4]. However, the detailed mechanisms remain unclear.

Evidence from previous imaging studies has demonstrated disruptions in brain metabolism, morphology and function in OSAHS patients with neurocognitive impairments [5–8]. There is a growing belief that the brain is composed of functional networks formed by dynamically connected regions that interact with each other to perform specific tasks [9], and a variety of neuropsychiatric disorders may be caused by dysfunction in these brain networks. An important development of brain networks was the discovery that different brain regions could exhibit synchronized blood oxygenation level dependent (BOLD) signal fluctuations in the "resting state" (when a subject is not performing any specific task) [10]. It has become an important research method in the field of cognitive neuroscience to explore the intra-network and inter-network functional connectivity through the synchronous spontaneous activity of different brain regions in resting state, which makes the brain resting-state networks (RSNs) become an important supplement to functional imaging technology. In the past decade, RSNs have been applied in the study of various neuropsychiatric diseases, including Alzheimer's disease, depression, and Parkinson's disease, and many characteristic manifestations have been found, which provide new insights for understanding the pathophysiological mechanisms of these diseases. For example, Zhang and coworkers [11] found that suicidal behavior of depressed adolescents may be related to abnormal functional connectivity in the default mode network (DMN), especially the abnormal connectivity in the precuneus/posterior cingulate cortex and left cerebellum may be a predictor of suicidal behavior of depressed adolescents. We hypothesized that cognitive impairment may be related to abnormal changes in RSNs in OSAHS patients, especially those RSNs involved in multiple cognitive processes, such as the DMN, executive control network (ECN), dorsal attention network (DAN), ventral attention network (VAN), somatomotor network (SMN), and salience network (SN).

At present, there are relatively few studies on dysfunction in brain RSNs in patients with OSAHS, and the results have been inconsistent. Zhang and coworkers [12] found that the functional connectivity of the anterior DMN, SMN and bilateral frontoparietal network decreased, and the functional connectivity of the posterior DMN increased. Li and coworkers [13] studied the functional connectivity of subregions within the default network of OSAHS patients, and found that the functional connectivity of right hippocampus, posterior cingulate gyrus, medial prefrontal lobe, and left middle temporal gyrus were all reduced. In addition, there have been no studies on the SN or attention networks in OSAHS patients. The aim of our study was to extend our understanding of brain dysfunction in patients with OSAHS. We compared the functional changes in the RSNs between OSAHS patients and healthy people. Furthermore, we evaluated the relationship between functional changes in RSNs, sleep breathing disorders and neurocognitive performance.

## Methods

### Participants

This study was performed in accordance with the Declaration of Helsinki and approved by the institutional ethical review committee, and informed consent was obtained from all participants. Eighteen never-treated OSAHS patients (age range, 25–65 years) and 18 healthy controls (age range, 25–65 years) were recruited. All participants were right-handed Han Chinese. The participants did not have other sleep disorders or uncontrolled cardiovascular disease, hypertension, or diabetes mellitus and had no current use of psychoactive medications. The Mini-Mental State Examination (MMSE) was used to exclude those with symptoms of cognitive deterioration (the participants with MMSE score < 24 were excluded) [14].

### Polysomnography

All OSAHS patients underwent polysomnography in the sleep center. For the 24 h before the examination, all patients were forbidden to take sleeping pills or drink alcohol, coffee, soda, strong tea and other energizing drinks. The sleep monitoring results were corrected and analyzed by specialized technicians. The apnea–hypopnea index (AHI), arousal index (ArI), saturation impair time below 90% during total sleep (SIT_90_), and sleep stages were recorded for our study.

### Neuropsychological assessments

The day after polysomnography, all participants underwent neuropsychological assessments, including the MMSE, trail-making test (TMT-A and B), digit span test (DST-forward and backward), and Rey's auditory verbal learning test (RAVLT-immediate recall, delayed recall, learning, and forgetting). Tests were administered and scored by an experienced doctor based on published procedures and lasted approximately 30 min.

### Functional magnetic resonance imaging (fMRI) data acquisition

The same day as the neuropsychological assessments, imaging data were acquired with a 3.0 T GE MRI scanner (GE Discovery MR750). The resting-state (rs)-fMRI data were acquired with an echo-planar imaging (EPI) sequence covering the whole brain, consisting of 250 sequential volumes (repetition time (TR) = 2000 ms, echo time (TE) = 30 ms, field of view (FOV) = 240 mm × 240 mm, matrix = 64 × 64, flip angle = 90°, slice thickness = 3.6 mm, 35 interleaved slices parallel to the bicommissural line). High-resolution structural T1-weighted images were also obtained (TR = 8.5 ms, TE = 3.3 ms, FOV = 240 mm × 240 mm, matrix = 256 × 256, flip angle = 12°, slice thickness = 1 mm, no gap, 184 sagittal slices). All participants were asked to lie flat with their eyes closed, breathe steadily, stay awake, and not do any specific thinking during fMRI scans. Earplugs or earphones were used to reduce noise, and sponge cushions were added on both sides of the head to reduce head movement.

### fMRI data preprocessing and independent component analysis (ICA)

fMRI data preprocessing was carried out with DPARSFA (http://rfmri.org/DPARSF) software. After transformation to the DICOM format, the first 10 time points of the rs-fMRI images were discarded, and slice timing, realignment, spatial normalization to the standard Montreal Neurological Institute (MNI) space, and spatial smoothing proceeded successively. Participants who had head motion > 2 mm in any dimension or angular rotation > 2° were excluded. The group spatial ICA was performed by a group ICA model for fMRI data (GIFT; http://icatb.sourceforge. net/) in three stages: The first stage was data reduction, in which a principal component analysis (PCA) was used to reduce individual fMRI data. The resulting volumes were concatenated and PCA was used again. After data reduction, the Infomax algorithm for ICA decomposition was used to identify the group components across all the participants. Finally, on the basis of the group components and the information found in the data reduction stage, the time courses and spatial maps for each participant were back-reconstructed, and the mean spatial maps for each component across all participants were displayed. Based on visual inspection and previous studies [15–18], we identified the RSNs we needed (including DMN, ECN, DAN, VAN, SMN, and SN) and saved them for subsequent analyses.

### Statistical analysis

Demographic and clinical characteristics, including age, years of education, sleep-disordered breathing parameters, and neuropsychological test scores, were analyzed using independent sample *t*-tests to compare OSAHS patients with healthy controls (*P* < 0.05 was deemed significant). Partial correlation analysis [12, 19] adjusted for age and education was performed to correlate sleep-disordered breathing parameters and neurocognitive performance. All the analyses were completed using the Statistical Package for the Social Sciences version 25.0 (SPSS, Chicago, IL, USA).

For functional imaging data, the analysis was performed using the SPM12 software, with age and education as nuisance covariates. we used a one-sample *t*-test to create a sample-specific component map as a spatial mask with the component maps for all participants (voxel-level familywise error (FWE)-corrected,* P* < 0.05). Then, a two-sample *t*-test was performed to explore between-group differences in the component time course-related activity within the spatial mask (cluster-level FWE-corrected,* P* < 0.05; single-voxel *P* = 0.001) and saved them as regions of interest (ROIs). Then, for patient group and control group, the average ICA z-scores of the ROIs were extracted using RESTplus software for further correlation analysis. The correlation analyses with clinical variables were performed using a partial correlation method (*P* < 0.05) in SPSS, the age and education were considered as the nuisance covariates.

## Results

### Demographic, clinical and neuropsychological findings

There were no significant differences in sex, age or years of education between the OSAHS patients and healthy controls, and the detailed demographic and clinical characteristics are shown in Table 1. As expected, OSAHS patients performed worse on neuropsychological tests, especially the TMT-A and RAVLT-delayed recall (Table 2). In addition, correlation analyses showed that RAVLT-learning scores were positively correlated with sleep events (REM + N3, %) (*r* = 0.67, *P* = 0.005) (Table 3).

**Table 1:** Demographic and clinical characteristics

	OSAHS (*n* = 18)	Healthy controls (*n* = 18)	*t*	*P*
Sex, M/F	16/2	16/2		
Age, yrs	44.2 ± 12.2	37.6 ± 10.4	1.76	0.087
Education, yrs	11.7 ± 2.8	13.7 ± 3.1	-1.83	0.077
AHI, per hour	73.1 ± 27.0	2.5 ± 0.8	11.08	< 0.001*
SIT_90_, %	40.0 ± 29.7	0.1 ± 0.1	5.70	< 0.001*
ArI, per hour	64.6 ± 30.4	4.6 ± 1.9	8.35	< 0.001*
REM + N3, %	14.7 ± 7.6	40.4 ± 5.5	-11.50	< 0.001*

Data are presented as average ± SD, *P < 0.05

*AHI* apnea–hypopnea index, *SIT*_*90*_ saturation impair time below 90%, *ArI* arousal index, *REM* rapid eye movement, *N3* nonrapid eye movement stage 3

**Table 2:** Neuropsychological data

Neuropsychological tests	OSAHS (*n* = 18)	Healthy controls (*n* = 18)	*t*	*P*
MMSE	27.5 ± 1.7	28.4 ± 1.2	-1.91	0.065
TMT-A	48.1 ± 16.6	36.4 ± 16.1	2.15	0.039*
TMT-B	118.9 ± 61.4	85.7 ± 36.8	1.97	0.057
DST-forward	8.1 ± 2.0	8.7 ± 1.1	-1.14	0.264
DST-backward	5.2 ± 1.8	5.2 ± 1.7	0.00	1.000
RAVLT-immediate recall	39.1 ± 8.0	43.8 ± 7.2	-1.84	0.074
RAVLT-delayed recall	6.7 ± 1.6	9.1 ± 2.7	-3.21	0.003*
RAVLT-learning	6.0 ± 1.4	6.4 ± 1.5	-0.80	0.429
RAVLT-forgetting	3.7 ± 1.7	2.8 ± 2.1	1.30	0.204

Data are presented as average ± SD, *P < 0.05

*MMSE* Mini-Mental State Examination, *TMT* trail-making test, *DST* digit span test, *RAVLT* Rey's auditory verbal learning test

**Table 3:** Correlation analyses between breathing disorders and neurocognitive performance in patients with OSAHS

	AHI		SIT_90_		ArI		REM% + N3%	
	*r*	*P*	*r*	*P*	*r*	*P*	*r*	*P*
MMSE	-0.18	0.495	-0.08	0.767	-0.17	0.524	0.07	0.801
TMT-A	-0.16	0.561	-0.05	0.867	-0.14	0.603	0.44	0.087
TMT-B	-0.25	0.357	-0.08	0.775	-0.12	0.652	-0.08	0.761
DST-forward	0.10	0.708	-0.02	0.957	0.14	0.615	-0.40	0.128
DST-backward	-0.20	0.452	-0.26	0.335	-0.08	0.782	-0.23	0.388
RAVLT-immediate recall	0.45	0.080	0.18	0.505	0.35	0.186	-0.44	0.091
RAVLT-delayed recall	0.18	0.499	-0.18	0.501	0.11	0.698	0.20	0.458
RAVLT-learning	-0.49	0.056	-0.04	0.876	-0.43	0.096	0.67	0.005*
RAVLT-forgetting	-0.26	0.332	0.09	0.740	-0.19	0.471	-0.13	0.638

*AHI* apnea–hypopnea index, *SIT*_*90*_ saturation impair time below 90%, *ArI* arousal index, *REM* rapid eye movement, *N3* nonrapid eye movement stage 3, *MMSE* Mini-Mental State Examination, *TMT* trail-making test, *DST* digit span test, *RAVLT* Rey's auditory verbal learning test, *P < 0.05

### rs-fMRI

**Fig. 1:**
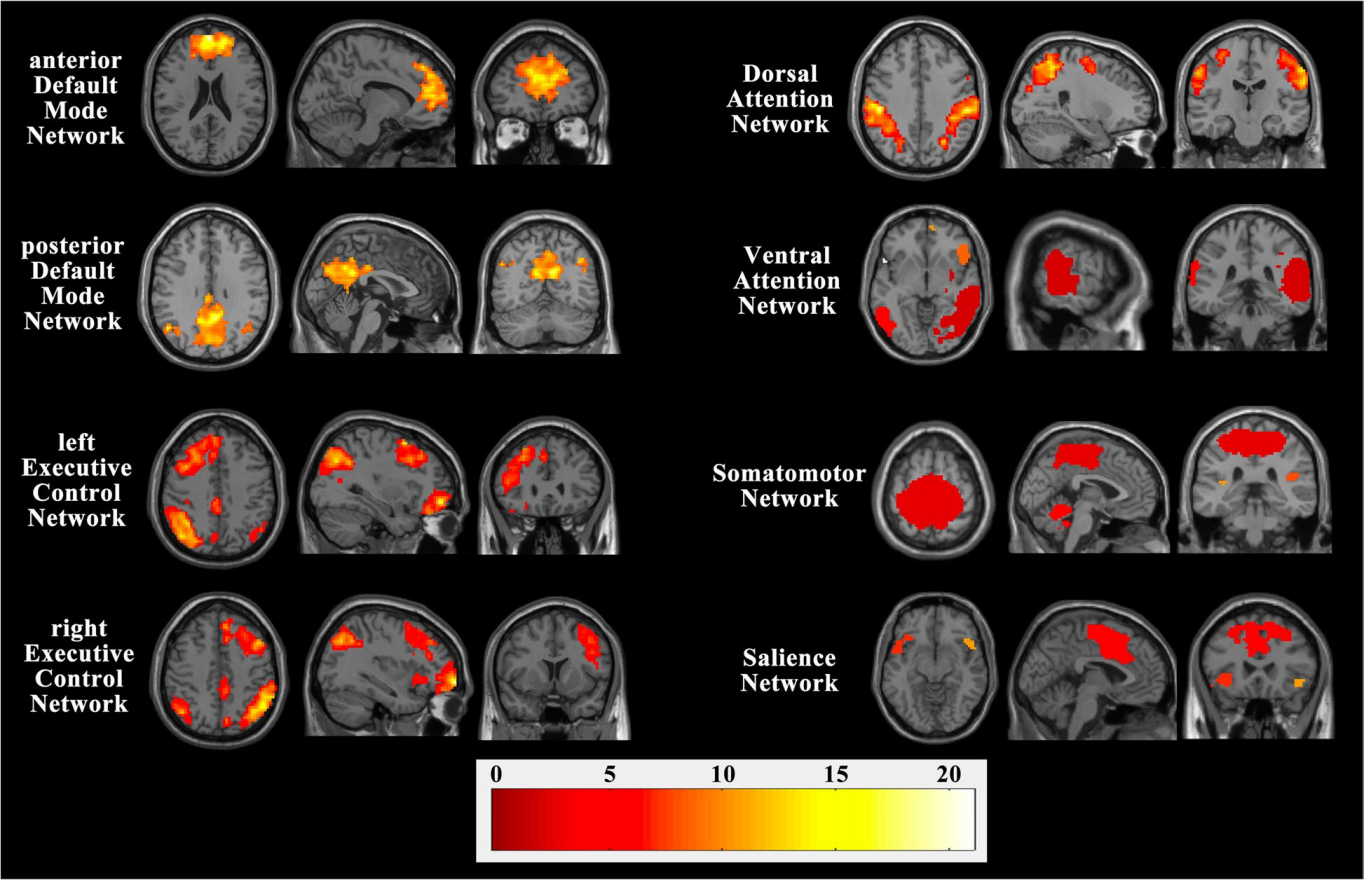
Spatial maps of cognitively and functionally relevant RSNs (one-sample *t*-test; voxel-level FWE-corrected, *P* < 0.05; cluster size threshold = 20 voxels)

**Fig. 2:**
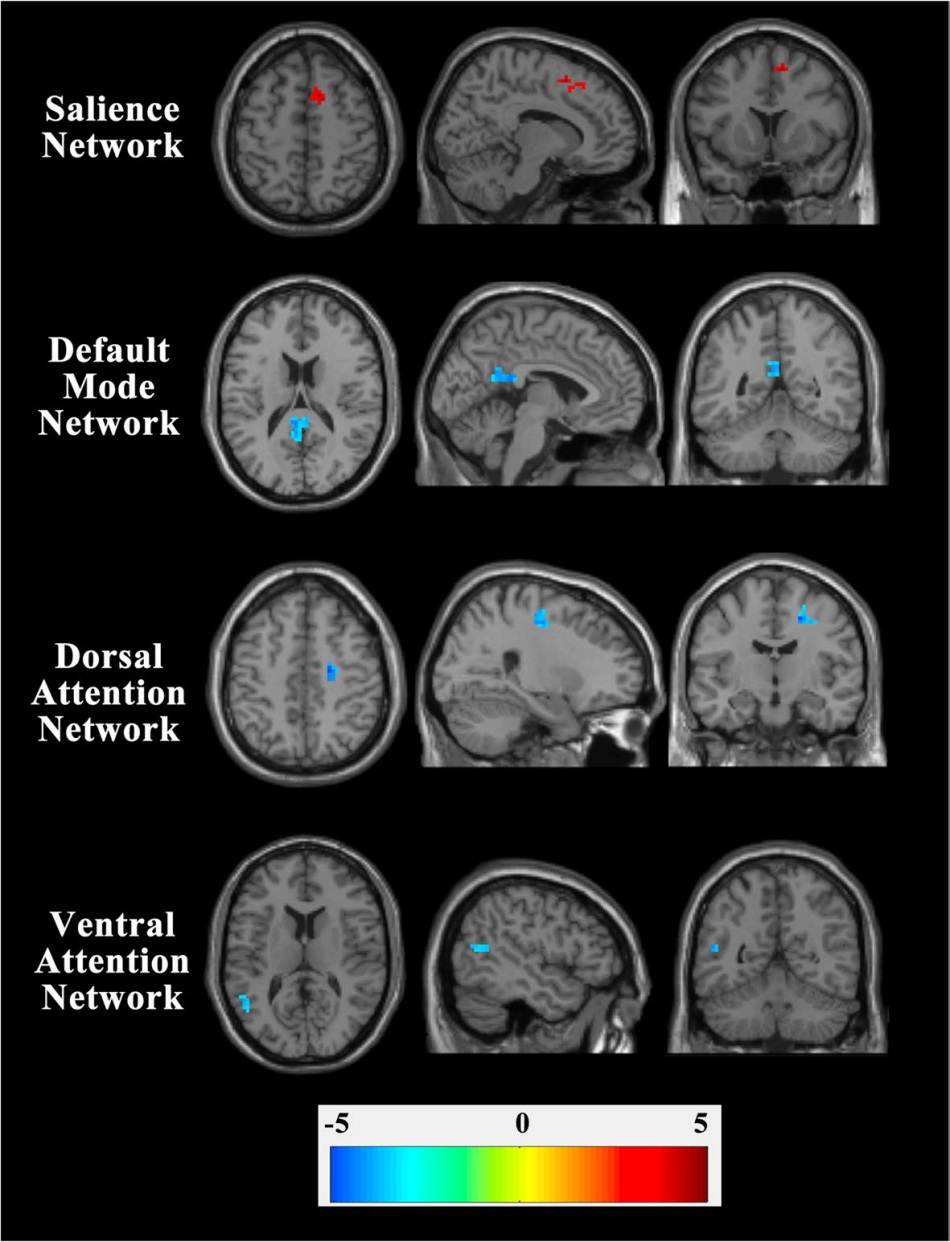
Contrasts of RSNs between OSAHS patients and healthy controls

Based on visual inspection, we characterized the following RSNs: DMN (anterior DMN and posterior DMN), ECN (left ECN and right ECN), DAN, VAN, SMN, and SN. The spatial maps of these cognitively and functionally relevant RSNs are shown in Fig. 1. Compared with the healthy controls, the patients with OSAHS displayed decreased component time course-related activity in the bilateral posterior cingulate gyrus (PCC) (x = -6; y = -48; z = 27) within the DMN, the right middle frontal gyrus (MFG) (x = 21; y = -12; z = 48) within the DAN, and the left superior temporal gyrus (STG) (x = -51; y = -57; z = 15) within the VAN. Moreover, significantly increased component time course-related activity was found in the right superior frontal gyrus (SFG) (x = 12; y = 12; z = 54) within the SN. Figure 2 shows the regions with differences of RSNs between OSAHS patients and healthy controls.

**Fig. 3:**
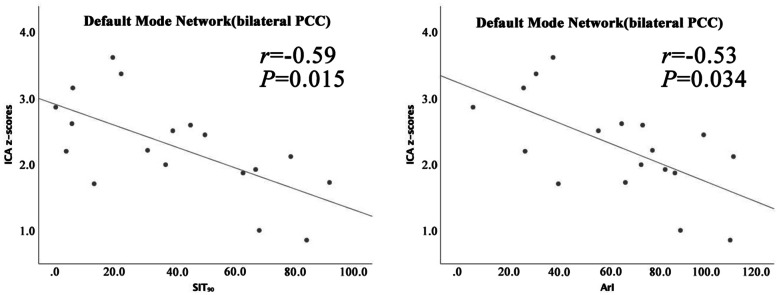
Correlation analyses between average ICA z-scores in the ROIs and measures of breathing disorders in patients with OSAHS. *PCC, posterior cingulate gyri; SIT*_*90*_*, saturation impair time below 90%; ArI, arousal index*

In the patients with OSAHS, correlation analyses showed that the average ICA z-scores in the bilateral PCC were negatively correlated with the SIT_90_ (*r* = -0.59, *P* = 0.015) and ArI (*r* = -0.53, *P* = 0.034) (Table 4, Fig. 3). There was no significant association between average ICA z-scores in the ROIs and clinical variables of the control group (Additional file 1: Table 5).

**Table 4:** Correlation analyses between average ICA z-scores in the ROIs and clinical variables in patients with OSAHS

	bilateral PCC(DMN)		right MFG(DAN)		left STG(VAN)		right SFG(SN)	
	*r*	*P*	*r*	*P*	*r*	*P*	*r*	*P*
MMSE	-0.19	0.489	0.09	0.746	0.19	0.475	-0.12	0.650
TMT-A	-0.32	0.220	-0.32	0.228	0.28	0.290	0.13	0.632
TMT-B	-0.04	0.880	-0.23	0.397	0.09	0.737	0.47	0.068
DST-forward	0.27	0.311	0.25	0.356	-0.07	0.807	-0.12	0.647
DST-backward	0.12	0.655	0.38	0.153	-0.05	0.863	0.06	0.837
RAVLT-immediate recall	0.09	0.742	0.11	0.699	0.08	0.762	0.00	0.990
RAVLT-delayed recall	-0.06	0.825	0.11	0.685	0.46	0.075	0.21	0.444
RAVLT-learning	-0.07	0.806	0.00	0.991	0.15	0.587	0.07	0.803
RAVLT-forgetting	0.12	0.667	0.02	0.957	-0.26	0.336	-0.11	0.686
AHI	-0.39	0.131	-0.21	0.437	0.08	0.781	0.04	0.899
SIT_90_	-0.59	0.015*	-0.38	0.153	0.05	0.845	0.14	0.609
ArI	-0.53	0.034*	-0.41	0.119	0.04	0.886	0.19	0.472
REM% + N3%	0.12	0.650	0.00	0.997	0.12	0.647	0.25	0.344

*AHI* apnea–hypopnea index, *SIT *_*90*_ saturation impair time below 90%, *ArI* arousal index, *REM* rapid eye movement, *N3* nonrapid eye movement stage 3, *MMSE* Mini-Mental State Examination, *TMT* trail-making test, *DST* digit span test, *RAVLT* Rey's auditory verbal learning test, *PCC* posterior cingulate gyri, *MFG* middle frontal gyrus, *STG* superior temporal gyrus, *SFG* superior frontal gyrus, * *P* < 0.05

## Discussion

Although the importance of cognitive dysfunction in patients with OSAHS is gaining increasing awareness, the occurrence and patterns of such impairments, the association between breathing disorders and cognitive features, and the functional brain changes and correlates have rarely been studied in detail. This study expanded our understanding of OSAHS and found that 1) patients performed worse on cognitive tests and 2) OSAHS affected the functional connection of brain RSNs, and this altered brain function revealed brain-behavior relationships.

The neuropsychological test results in this study demonstrated impairments in memory, attention, and executive functions in OSAHS patients, which is consistent with previous studies. Mazza and coworkers [20] found that compared with controls, OSAHS patients performed poorly in all alertness and attention tests at any time during the day. Other studies [21, 22] also found that OSAHS patients suffered from impaired executive function, including reduced information processing speed, reaction ability and problem solving ability.

Repeated microarousals or complete awakenings at night in OSAHS patients can disrupt the normal balance of the sleep internal environment. The results of the present study suggested that the nocturnal sleep structure in the OSAHS group was disturbed, with a significantly greater proportion of N1 and N2 sleep periods and a reduced or even absent proportion of N3 and REM sleep periods. The N3 sleep period, also known as slow-wave sleep (SWS), is an important factor in declarative memory consolidation and enhancement, and studies have shown that a reduction in SWS may inhibit neurogenesis and synaptic plasticity, leading to cognitive impairment [23, 24]. It has also been found that both SWS and REM sleep periods contribute to the consolidation of selective memory [25]. Correlation analyses in our current study showed that RAVLT-learning scores were positively correlated with sleep events (REM + N3, %), suggesting that reduced REM and N3 sleep periods may be an important factor for the decline in episodic memory and learning abilities.

Eight RSNs were identified by applying ICA that were spatially consistent across subjects and corresponded to functionally related patterns. Statistical analysis of the imaging data demonstrated that compared to the healthy controls, OSAHS patients exhibited functional connectivity within the DMN, DAN and VAN and increased functional connectivity within the SN. The DMN, SN, DAN and VAN are large-scale brain RSNs that play a crucial role in cognitive information processing, and the abnormal functional connections in the PCC, MFG, STG and SFG suggests that these impaired regions may be an indirect cause of the cognitive deficits observed in patients with OSAHS.

The DMN has been associated with introspection, emotional regulation and episodic memory processing [26, 27]. Intermittent hypoxia caused by OSAHS is one of the important factors for neuronal apoptosis [23, 24], especially highly metabolically active clusters, such as the PCC, one of the most active brain regions in the resting state [28]. The negative correlation between PCC activity and SIT_90_ in our study suggested this. The PCC is a key node in the posterior DMN that has extensive connections with medial structures in the temporal lobe, such as the parahippocampal gyrus and entorhinal cortex, and is mainly involved in the processing of consciousness and memory [26]. Li and coworkers [29] found that OSAHS patients showed dysfunction in the bilateral PCC cluster by the amplitude of low-frequency fluctuation (ALFF) method and correlations with cognitive impairment. Furthermore, our study found a negative correlation between the average ICA z-scores in the bilateral PCC and ArI events in our study revealed that the functional damage in the PCC may have associations with frequent microarousals.

There are two main attentional networks thought to contribute to movement execution: the DAN is mainly involved in executive processes related to internal spatial attention, continuous motion planning and visual working memory movement, and the VAN is usually activated at the time of unexpected exposure to stimuli and thus may be involved in the detection of unconscious or unexpected stimuli and the triggering of attention shifts [30, 31]. The impaired regions associated with the DAN and VAN were contained in the key nodes of these attention networks, especially the right MFG, which has been suggested to be a 'circuit breaker', a region that links the DAN and VAN and interrupts ongoing neural activity in the DAN to reorient one's attention to an external stimulus [32].

The SN is a limbic-paralimbic network that is primarily composed of the anterior insula, temporoparietal junction, dorsal anterior cingulate cortex, and frontal opercular cortex, is mainly involved in adaptively guiding attention and behavior, and constitutes a critical interface between cognitive, affective, motivational, and homeostatic systems [33]. In this study, we found that functional connectivity of the right SFG within the SN increased. Although the exact mechanisms underlying the processes leading to cortical overactivation remain unknown, these increased responses have been explained as a 'neural compensations' for limiting the progression in cognitive decline that would have required an increased 'effort' to achieve a normal level of behavioral performance [34].

Notably, Zhang and coworkers [12] found that functional connectivity of the DMN (PCC) in OSAHS patients was increased, which was inconsistent with our study. This may have been due to differences in disease severity in the two study samples: the AHI for the OSAHS group in Zhang's study was 54.7 ± 19.9, but the OSAHS group included in our study was more severe (AHI: 73.1 ± 27.0). Therefore, the PCC in our study may have been beyond the compensation stage, resulting in a decrease in functional connectivity.

One main limitation of our study was that the sample size was relatively small, which may have affected the accuracy of the research results, and therefore, the sample size should be increased in future studies to group participants and conduct longitudinal analyses. Another limitation was that partial correlations only assessed within-group associations, and group comparisons or conclusions could not be made from the present study. Furthermore, this study mainly discussed the functional changes in brain RSNs without considering the interactions between brain structure, function and metabolism.

## Conclusions

The present study was able to demonstrate that the DMN, SN, DAN, and VAN are impaired during the resting state, which was associated with decreased performance in functionally distinct aspects of cognition in patients with OSAHS, and the intermittent hypoxia and sleep fragmentation caused by OSAHS are likely to be the main influencing factors. Future investigations can explore the brain impairments in OSAHS patients using multimodal imaging techniques.

## Supplementary Information

**Additional file 1: Table 5. **Correlation analyses between average ICA z-scores in the ROIs and clinical variables in healthy controls.

## Data Availability

The datasets used during the current study are not publicly available due to reasons of sensitivity but are available from the corresponding author on reasonable request.

## Abbreviations

AHI: Apnea–hypopnea index
ALFF: Amplitude of low-frequency fluctuation
ArI: Arousal index
BOLD: Blood oxygenation level dependent
DAN: Dorsal attention network
DMN: Default mode network
DST: Digit span test
ECN: Executive control network
EPI: Echo-planar imaging
FWE: Familywise error
ICA: Independent component analysis
MFG: Middle frontal gyrus
MMSE: Mini-Mental State Examination
MRI: Magnetic resonance imaging
N3: Nonrapid eye movement stage 3
OSAHS: Obstructive sleep apnea–hypopnea syndrome
PCA: Principal component analysis
PCC: Posterior cingulate gyri
RAVLT: Rey's auditory verbal learning test
REM: Rapid eye movement
ROIs: Regions of interest
RSNs: Resting-state networks
SFG: Superior frontal gyrus
SIT_90_: Saturation impair time below 90%
SMN: Somatomotor network
SN: Salience network
STG: Superior temporal gyrus
SWS: Slow-wave sleep
TE: Echo time
TMT: Trail-making test
TR: Repetition time
VAN: Ventral attention network
