# Supplementary material for: Preliminary study on brain resting-state networks and cognitive impairments of patients with obstructive sleep apnea–hypopnea syndrome
Source: BMC Neurol. 2022 Dec 7;22:456. doi: 10.1186/s12883-022-02991-w (PMC9728000; doi:10.1186/s12883-022-02991-w)
Supplement: Supplementary file 1 — Additional file 1: Table 5. Correlation analyses between average ICA z-scores in the ROIs and clinical variables in healthy controls. [file 12883_2022_2991_MOESM1_ESM.docx]

**Table 5** Correlation analyses between average ICA z-scores in the ROIs and clinical variables in healthy controls

|  | bilateral PCC(DMN) | |  | right MFG(DAN) | |  | left STG(VAN) | |  | right SFG(SN) | |
| --- | --- | --- | --- | --- | --- | --- | --- | --- | --- | --- | --- |
|  | *r* | *P* |  | *r* | *P* |  | *r* | *P* |  | *r* | *P* |
| MMSE | -0.49 | 0.053 |  | -0.13 | 0.629 |  | 0.48 | 0.059 |  | 0.27 | 0.308 |
| TMT-A | 0.34 | 0.202 |  | 0.33 | 0.213 |  | -0.40 | 0.121 |  | -0.02 | 0.948 |
| TMT-B | 0.38 | 0.145 |  | 0.23 | 0.386 |  | -0.35 | 0.186 |  | -0.09 | 0.751 |
| DST-forward | -0.19 | 0.473 |  | -0.33 | 0.215 |  | 0.30 | 0.267 |  | 0.44 | 0.090 |
| DST-backward | -0.42 | 0.104 |  | -0.11 | 0.672 |  | 0.30 | 0.253 |  | 0.32 | 0.226 |
| RAVLT-immediate recall | 0.02 | 0.934 |  | -0.22 | 0.417 |  | -0.27 | 0.314 |  | -0.01 | 0.961 |
| RAVLT-delayed recall | -0.25 | 0.354 |  | -0.06 | 0.822 |  | -0.05 | 0.848 |  | -0.08 | 0.781 |
| RAVLT-learning | 0.34 | 0.201 |  | -0.13 | 0.620 |  | 0.11 | 0.695 |  | -0.05 | 0.862 |
| RAVLT-forgetting | 0.24 | 0.366 |  | -0.34 | 0.203 |  | 0.10 | 0.707 |  | 0.11 | 0.675 |
| AHI | -0.36 | 0.174 |  | -0.47 | 0.068 |  | 0.42 | 0.109 |  | 0.19 | 0.489 |
| SIT_90_ | -0.36 | 0.178 |  | -0.35 | 0.181 |  | 0.48 | 0.061 |  | 0.35 | 0.188 |
| ArI | -0.19 | 0.479 |  | -0.46 | 0.071 |  | 0.33 | 0.216 |  | 0.11 | 0.697 |
| REM%+ N3% | 0.30 | 0.256 |  | 0.48 | 0.061 |  | 0.37 | 0.157 |  | 0.10 | 0.724 |

*AHI, apnea-hypopnea index; SIT_90_, saturation impair time below 90%; ArI, arousal index; REM, rapid eye movement; N3, nonrapid eye movement stage 3; MMSE, Mini-Mental State Examination; TMT, trail-making test; DST, digit span test; RAVLT, Rey's auditory verbal learning test; PCC, posterior cingulate gyri; MFG, middle frontal gyrus;* *STG, superior temporal gyrus;* *SFG, superior frontal gyrus*
